# Impeding Quorum Sensing Among the Intestinal Microbiota Impacts the Metastatic Rate of Colorectal Cancer

**DOI:** 10.1002/cam4.71009

**Published:** 2025-06-19

**Authors:** Matthew Dietz, Travis J. Gates, Rakesh Sikdar, Subbaya Subramanian, Mikael H. Elias, Christopher Staley

**Affiliations:** ^1^ Division of Basic and Translational Research, Department of Surgery University of Minnesota Minneapolis Minnesota USA; ^2^ BioTechnology Institute, University of Minnesota St. Paul Minnesota USA; ^3^ Department of Pharmacology University of Minnesota Medical School Minneapolis Minnesota USA; ^4^ Department of Biochemistry, Molecular Biology, and Biophysics University of Minnesota St. Paul Minnesota USA; ^5^ Masonic Cancer Center, University of Minnesota Minneapolis Minnesota USA

**Keywords:** autoinducer, microbiome, organoid, quorum quenching, signaling

## Abstract

**Background:**

The gut microbiota is associated with colorectal cancer (CRC) risk and CRC metastatic potential. However, the role of bacteria in CRC progression and metastasis remains unclear.

**Aims:**

Here, we hypothesized that microbial communication, mediated through quorum sensing (QS), was a critical component regulating microbial functions related to cancer progression and metastasis.

**Materials & Methods:**

To test this, male and female C57BL/6 mice were injected with organoids modeling aggressive colon cancer (CRC), carrying mutations in *Apc*, *Kras*, *p53*, and *Smad4*. Two groups of mice were treated with two different quorum quenching (QQ) lactonases (GcL or SsoPox) for 8 weeks (*n* = 10/group/sex). Fecal samples were collected weekly and characterized by Illumina next‐generation sequencing, with tissues collected during necropsy.

**Results:**

Male mice treated with SsoPox had fewer metastases than control mice (*χ* ^2^ = 3.206, *p* = 0.073), with no SsoPox‐treated male developing a metastasis. In contrast, female mice treated with SsoPox had more metastases than control mice (*χ* ^2^ = 2.554, *p* = 0.110), and every female, SsoPox‐treated mouse that developed a primary tumor also developed metastasis by the experimental endpoint. However, QQ treatment was shown to minimally affect the gut microbiome composition. Similarly, no significant differences were observed in inflammatory response as assessed by immunofluorescent staining or fecal concentrations of immunoglobulin A, calprotectin, or lipocalin‐2. Differences in fecal short‐chain fatty acid concentrations also did not differ significantly.

**Discussion:**

These results suggest that QQ treatment has a sex‐based effect on CRC metastatic rate.

**Conclusion:**

Targeting communication among the gut microbiome may be a promising avenue for the development of CRC therapies that minimally impact microbial community composition and host immune response.

## 1 Introduction

Colorectal cancer (CRC) is the third most common cancer worldwide, with a 60% increase in prevalence predicted between 2020 and 2040 [1]. Even with standard, first‐line therapies for CRC, most patients' tumors will progress within a year [2]. Metastasis in CRC is a grim marker of patient outcome, with 5‐year survival rates plummeting from 90% for localized disease to 14% in metastatic disease [3, 4]. A complete understanding of the mechanisms driving CRC metastasis is still being developed, but some intestinal microbes such as *Bacteroides fragilis, Prevotella* spp., and *Fusobacterium nucleatum* have been associated with both CRC risk and CRC metastatic potential [5, 6]. Therefore, the microbiome likely serves as a novel target for CRC treatment, including as an anti‐metastatic treatment, and modulation of the microbiome has already shown some success in improving the outcomes of other cancer therapies [7, 8].

Gut bacteria communicate with one another, as well as their host, through quorum sensing (QS), or the production and detection of autoinducer molecules [9, 10]. Both interspecies and intraspecies communication via QS allow bacteria to synchronously alter their function based on population densities, allowing them to maximize benefits from collective functions that would be too costly in low‐density communities [9, 10, 11]. *N*‐acyl homoserine lactones (AHLs) are a common group of QS molecules that are produced predominantly by Gram‐negative bacteria [12, 13]. The number and diversity of QS molecules, including AHLs, are not well understood, but studies on AHLs in cancer have shown AHLs to have microenvironment‐dependent effects on human cancer cells and that QS can promote CRC metastasis in an orthotopic mouse model [14, 15]. While exact mechanisms of action have yet to be outlined, these initial findings demonstrate the potential of microbiome‐targeted therapies to impact human disease.

Gram‐positive bacteria degrade AHLs by expelling quorum quenching (QQ) enzymes such as lactonases and acylases [16], which is thought to give them an advantage by disrupting the ability of Gram‐negative bacteria to communicate and coordinate functions such as biofilm and virulence factor production [12, 17, 18]. Instead of AHLs, QS signaling in Gram‐positive bacteria relies on an autoinducer‐2, leaving them unhindered by QQ AHL degradation [19]. At normal physiological levels, this leads to a natural homeostasis between QS and QQ systems within most microbiomes, including the gut [19]. Supraphysiological doses of QQ enzymes have shown promising results as a way to reduce biofouling and pathogenic infections in fisheries and poultry farms without the use of antibiotics [20, 21], but research into their potential use in modifying the host microbiome to modulate its role in other diseases has remained largely uninvestigated [22, 23].

SsoPox, a thermostable lactonase, is active primarily on longer‐chain AHLs (> C8–C12), while another lactonase, GcL, has a much broader substrate specificity compared to SsoPox, acting on C4‐C12 AHLs [18, 24]. Both SsoPox and GcL have demonstrated the ability to inhibit biofilm production and reduce the production of virulence factors, including in vivo in the case of SsoPox [18, 20, 24, 25, 26]. We elected to study two distinct lactonases, as bacteria produce species‐specific AHLs, the acyl side chains of which differ in both length and chemical structure [27, 28]. Thus, lactonases with distinct substrate preferences may allow for precision QQ treatment to target specific bacterial species thought to contribute to pathogenesis without disrupting the activity of others [17, 18, 23, 24].

Our objective was to determine if QQ lactonase treatments could modify the QS behavior of the gut microbiome, resulting in slower tumor growth or reduced metastatic burden in aggressive CRC. We hypothesized that QQ treatment would affect the rate of CRC metastasis without significantly changing the gut microbiome composition, potentially by modulating host inflammatory or immune response.

## 2 Methods

### 2.1 Mice

C57BL/6 mice were purchased at 35–41 days of age from Jackson Labs and given drinking water supplemented with SsoPox or GcL (both at 1 mg/mL) [29] or a drinking water control (DW), which was supplied ad lib (*n* = 10/group). Mice were individually housed in conventional housing [30]. Fecal pellets were collected weekly and stored at −20°C until DNA extraction. At the end of eight weeks or when moribund, mice were sacrificed, tissues were collected, and tumor and metastatic burden were assessed by visual inspection. The experiment was approved by the University of Minnesota Institutional Animal Care and Use Committee (IACUC), protocol 2212‐40606A.

### 2.2 Organoid Preparation, Injection, and Monitoring

We used the AKPS organoid derived from C57BL/6 mice, which contains driver gene mutations in *Apc, Kras, p53*, and *Smad4* (AKPS) [31, 32], to model aggressive CRC. Organoids were cultured in 10% Matrigel media before being injected into the colon wall, with each mouse receiving two 5000‐cell injections [31]. Following the injection, mice were monitored for eight weeks.

### 2.3 Lactonase Preparation

We focus on the lactonases SsoPox and GcL because these enzymes represent two major classes of lactonases—phosphotriesterase‐like lactonase and metallo‐beta‐lactamase‐like lactonase, respectively. Both enzymes are extremely thermally stable [33, 34], and this critical property typically correlates with high resistance toward harsh conditions, including protease resistance as observed for SsoPox [35], key properties that are likely essential to enable these enzymes to remain active through the digestive system. Moreover, these two enzymes possess distinct AHL substrate preferences: SsoPox preferentially hydrolyzes long‐chain AHLs (C8 or higher), while GcL is a broad‐spectrum lactonase [36].

Specifically, the mutant lactonase SsoPox W263I, referred to as SsoPox throughout this article, that is improved for activity [34] and wild‐type GcL with an N‐terminal Strep‐tag II [24] were overexpressed in *Escherichia coli* strain BL21 Star (DE3) (Invitrogen, Carlsbad, CA, USA) containing the pGro7 plasmid (TakaRa Bio, San Jose, CA, USA). The enzymes were produced using a 75‐L fermentation system (New Brunswick Scientific, Edison, NJ, USA) operated by the University of Minnesota BioResource Center and purified as previously described [33]. The purification protocol takes advantage of the high thermal stability of both enzymes [34]. Briefly, cell lysates were centrifuged, and the supernatants were subjected to heat treatment to precipitate host cell contaminants at 75°C (for SsoPox W263I) and 65°C (for GcL) for 30 min. Precipitated contaminants were removed by centrifugation (15,000 *g*/30 min/4°C) and the supernatants were ultrafiltered (0.6 μm for GcL; 0.6 μm followed by 0.2 μm nominal filter for SsoPox), concentrated, and lyophilized. Both enzyme preparations were assayed for quality and purity using SDS‐PAGE (6%–12%) and tested for activity against 5‐thiobutyl‐γ‐butyrolactone (TBBL) substrate (synthesized by Enamine LTD, Kyiv, Ukraine) in an activity buffer (50‐mM HEPES pH 8.0, 150‐mM NaCl, 0.2‐mM CoCl_2_) containing 0.5‐mM TBBL and 1‐mM Ellman's reagent (5,5′‐dithiobis‐[2‐nitrobenzoic acid] or DTNB) as previously described [37]. The specific activity for TBBL of the purified proteins was 28,410 and 7896 μmol min^−1^ mg^−1^ of lyophilized enzyme for SsoPox W263I and GcL, respectively.

### 2.4 DNA Extraction and Sequencing

DNA was extracted from single, thawed mouse pellets (~100 mg) using the DNeasy PowerSoil Pro kit (QIAGEN, Hilden, Germany) on the QIAcube platform. The V4 region of the 16S rRNA gene was amplified using the 515F/806R primer set [38] by the University of Minnesota Genomics Center (UMGC). Paired‐end sequencing was done on the Illumina MiSeq platform (Illumina Inc, San Diego, CA, USA) at a read length of 301 nucleotides by UMGC [39]. Sequences were processed using Mothur and our previously published pipeline [30]. Briefly, sequences were aligned against the SILVA database (ver. 138.1) [40] and clustered at 99% sequence similarity. Samples were rarefied to 5000 sequence reads by random subsampling, and taxonomic classification was performed against the Ribosomal Database Project (ver. 18) [41]. Sequencing data were deposited in the National Center for Biotechnology's Sequence Read Archive under BioProject accession number SRP477359.

### 2.5 Characterization of Functional Response

Immunofluorescence staining for CD163, CD206, and CD68 was performed on tumor tissue to quantify immune infiltrates, primarily T cells and macrophages. Assays for immunoglobulin A (IgA), calprotectin, and lipocalin 2 (Lcn‐2) were performed using enzyme‐linked immunosorbant assays (ELISAs) from Abcam (Cambridge, UK).

Quantification of short‐chain fatty acids (SCFAs; 2‐methylbutyric acid, acetic acid, butyric acid, isobutyric acid, isovaleric acid, propionic acid, and valeric acid) was performed by the Center for Metagenomics and Proteomics using a previously described method with modifications [42]. Briefly, feces were lyophilized and extracted using a 0.5% aqueous phosphoric acid solution at a ratio of 0.1 g material to 1 mL. Samples were homogenized using a Precellys bead beater homogenizer (four 10 s pulses at 15,000 × *g*, 20 s pause, 0°C), after which the organic phase was stored in a fresh vial. Analytical assessment was performed using a gas chromatography‐coupled mass spectrometry (GC–MS) platform. The MS detector was a high‐resolution Agilent 7200 QTOF instrument operated in electron impact ionization mode at 70 eV electron energy that scanned from 35 to 350 *m/*z. Quantification of SCFAs from the raw GC–MS data was performed using the open‐source software Skyline [43]. Calibration curves were fit independently for each compound by linear regression using the peak ratio of each compound to the global internal standard. No regression weighting was used, and regression intercepts were forced through zero. All calibration curves were fit with an *R* ^2^ of at least 0.995 precision.

### 2.6 Statistics

Shannon and Chao1 indices were calculated using (version 1.41.1) [44]. Also in Mothur, beta diversity was evaluated based on Bray‐Curtis distances using analysis of similarity [45] (ANOSIM), and ordination was performed by principal coordinate analysis (PCoA). Differences in alpha diversity and analyte concentrations were determined by analysis of variance (ANOVA) using Tukey's *post‐hoc* test. Relative abundances of taxa were compared among groups with Kruskal–Wallis analysis. Differences in the rate of metastasis between groups were determined with Chi‐squared tests. Statistics were calculated using XLSTAT ver. 2022.1 1.1243, Excel ver. 16.0 (Addinsoft, Belmont, MA). All statistics were evaluated at *α* = 0.05, with Bonferroni correction for multiple comparisons.

## 3 Results

**FIGURE 1:**
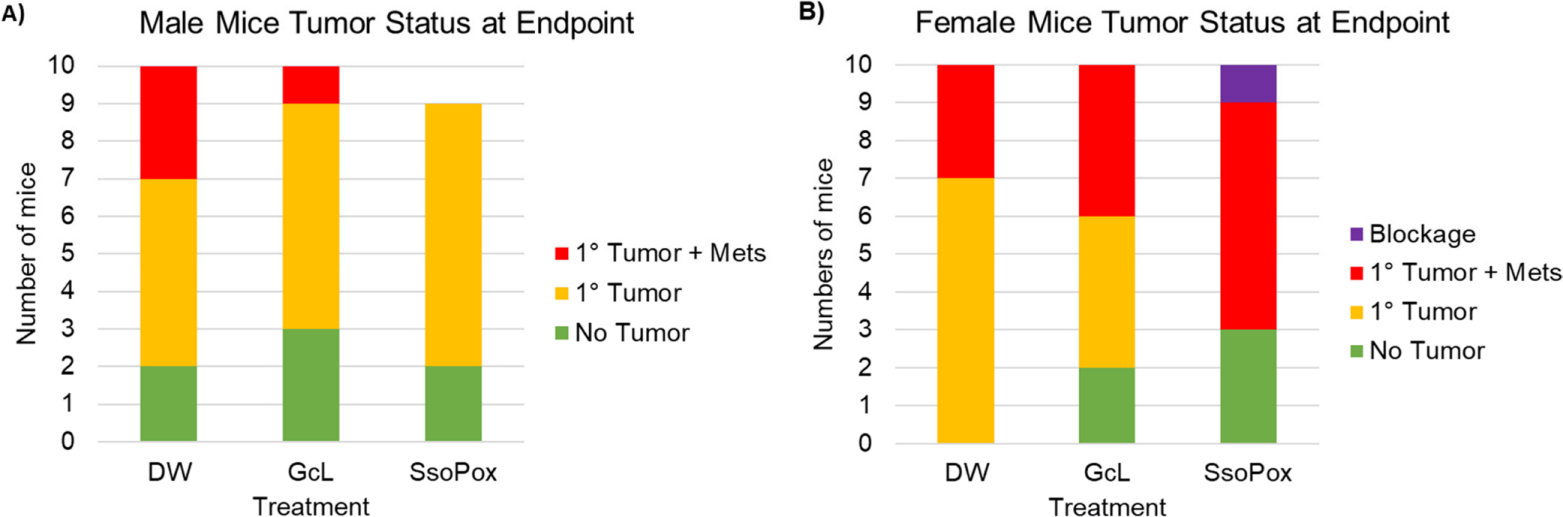
Bar graph of male (A) and female (B) disease progression at the time of necropsy. One male SsoPox mouse died from complications of organoid injection. One female SsoPox mouse developed a blockage at week three that required euthanasia.

In both male and female mice, there were no significant differences in the rate of organoid engraftment between treated and untreated mice (80%, 70%, and 78% for SsoPox, GcL, and control males, respectively; 100%, 80%, and 70% for females; ANOVA *p* = 1.000, 0.140, Figure 1). SsoPox‐treated males had a lower rate of metastasis (0.00%) when compared to controls (30% for controls; *χ* ^2^ = 3.206, *p* = 0.073, Figure 1A); however, in females, SsoPox treatment resulted in more metastases than untreated females (60% vs. 30%, *χ* ^2^ = 2.554, *p* = 0.110, Figure 1B).

### 3.1 QQ Lactonase Treatments Minimally Affect the Microbiome

There were no differences in alpha diversity, measured using the Shannon and Chao1 indices, among treatment groups in male mice (ANOVA *F* = 0.222, 0.372, *p* = 0.803, 0.694). Similarly, there were no differences among treatment groups in female mice (ANOVA *F* = 0.610, 0.278, *p* = 0.553, 0.761). Over the eight‐week period, the Shannon index increased in males but did not significantly increase in females (ANOVA *F* = 6.345, 2.775, *p* = 0.015, 0.102). In that same time, the Chao1 index did not significantly change in either sex (ANOVA *F*s < 0.113, *p* > 0.739).

**FIGURE 2:**
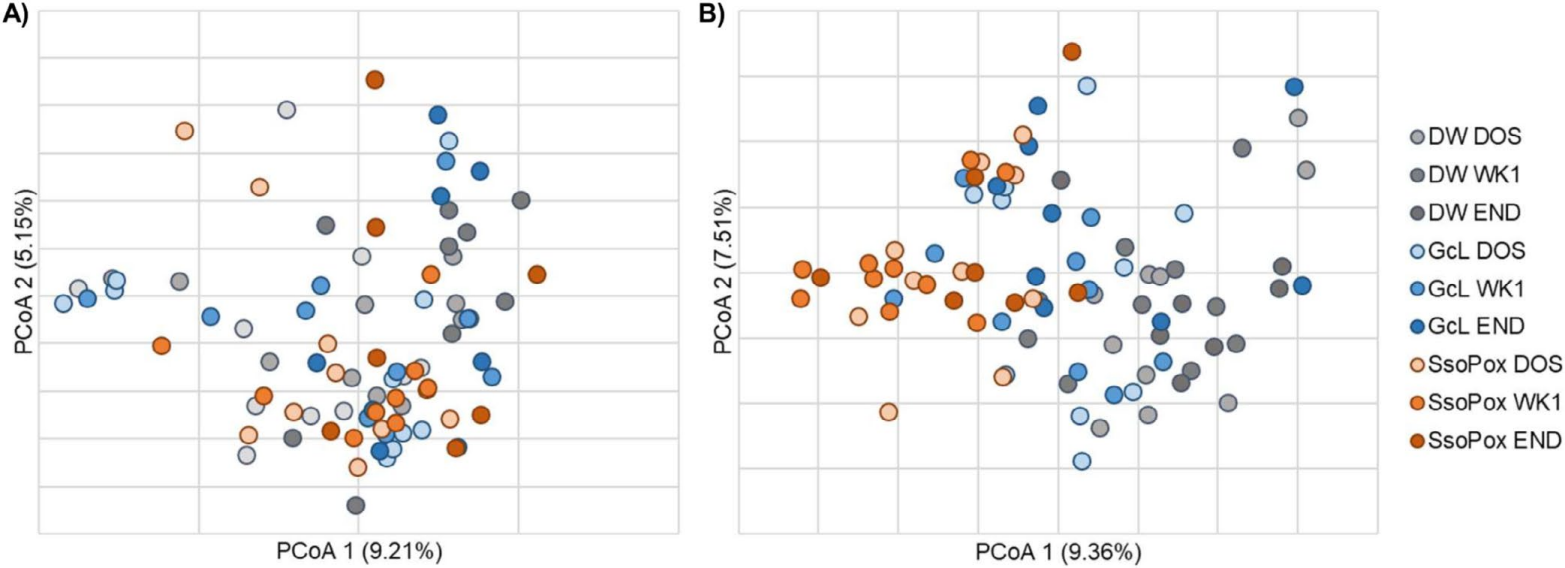
Principal coordinate analysis (PCoA) of male (A) and female (B) beta diversity on the day of organoid injection (DOS), end of week one (WK1), and experimental endpoint (END) for all treatment groups.

**FIGURE 3:**
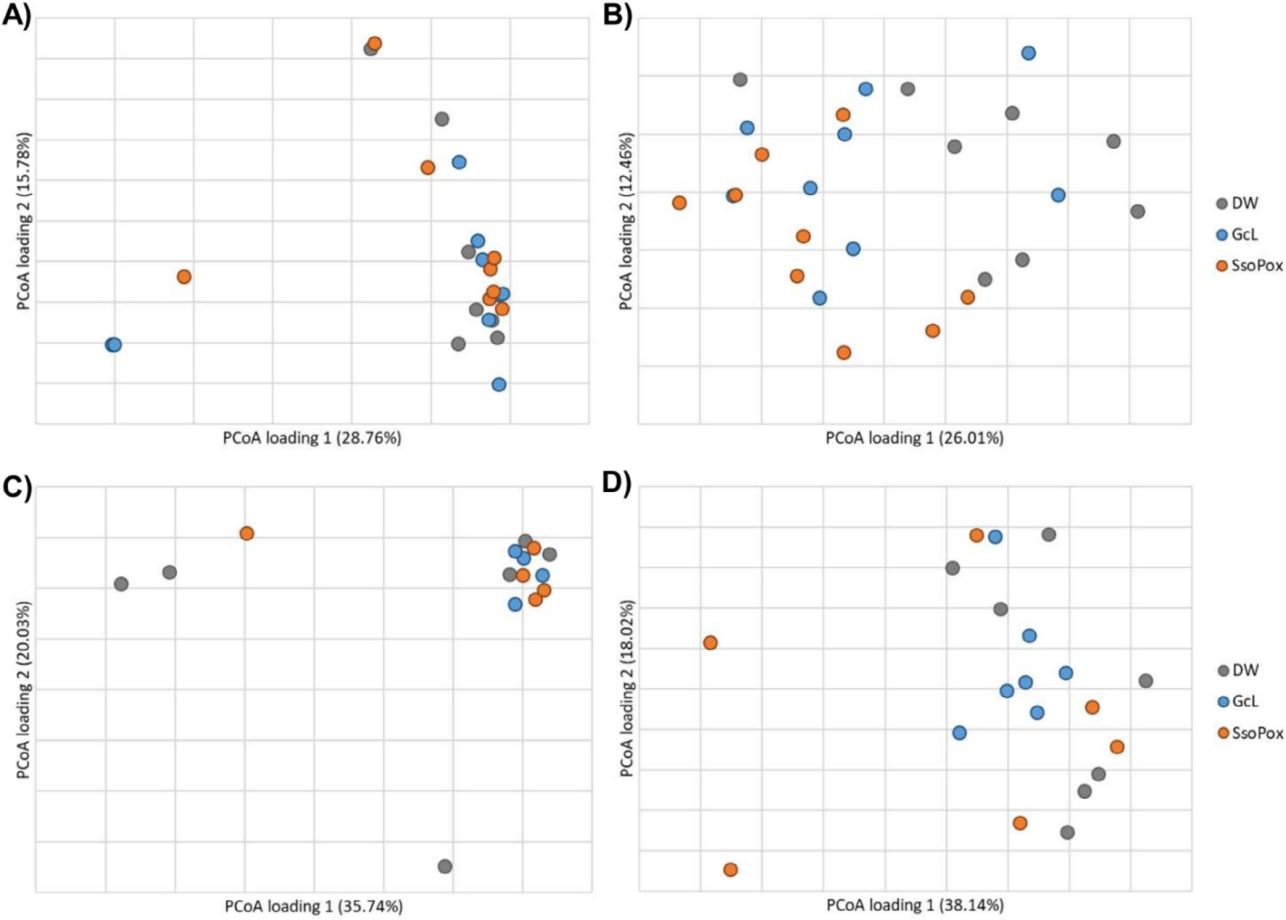
Principal coordinate analysis (PCoA) of beta diversity from colon (A and B) and tumor (C and D) samples in males (A, C) and females (B, D).

The gut microbiome community structures (β‐diversity) of male mice were not significantly different among treatment groups at endpoint (ANOSIM *R* = −0.005, *p* = 0.481, Figure 2A). *Bacteroides* was significantly more abundant in the male SsoPox‐treated mice at endpoint when compared to controls (Kruskal–Wallis *K* = 7.621, *p* = 0.022). Male mice that developed tumors had greater relative abundances of *Clostridiales* (unable to classify at greater resolution) and lower abundances of *Lactobacillus* when compared to those that did not develop tumors (*K* = 4.234, 4.747, *p* = 0.040, 0.029), as has been previously reported in both mice and humans [46, 47, 48]. Among predominant genera, none showed differential abundances based on the presence of metastasis. Even in the adherent microbiome, there were no significant differences in male samples by treatment group in either colon or tumor samples (ANOSIM *R* = −0.030, −0.055, *p* = 0.732, 0.748; Figure 3A,C).

The gut microbiome compositions of female mice differed among all groups at both baseline and endpoint (ANOSIM *R* = 0.268, 0.200, *p* = 0.001, 0.008, respectively; Figure 2B), suggesting differences between groups were associated with their baseline composition, irrespective of treatment. However, *Romboutsia* was found to be significantly less abundant in SsoPox‐treated mice when compared to controls at endpoint (*K* = 6.657, *p* = 0.036). Similar to male mice, females that developed tumors had greater relative abundances of *Clostridiales* (not further classified) and *Alistipes*, a genus associated with inflammation in CRC [49, 50], when compared with those that did not develop tumors (*K* = 4.48, 4.31, *p* = 0.034, 0.038). Female mice that developed metastases had significantly greater abundances of *Ruminococcaceae* (not further classified) than those that did not metastasize by experiment endpoint, though this family has been found to be associated with eubiosis and microbiome restoration after dextran sodium sulfate (DSS)‐induced bowel inflammation [51, 52]. In the female adherent microbiome in healthy colon samples, there was a significant difference between SsoPox and control mice (ANOSIM *R* = 0.432, 0.001), and SsoPox mice had significantly higher abundances of *Muribaculaceae* (not further classified) (Figure 3B). For female adherent tumor microbiomes, there was an overall difference in the compositions of the treatment groups (ANOSIM *R* = 0.125, *p* = 0.036), but none of the individual comparisons reached significance, and no genera with a relative abundance > 1% were significantly different between treatment groups (Figure 3D).

### 3.2 QQ Lactonases Treatments Do Not Induce an Immune Response or Microbial SCFA Production

**FIGURE 4:**
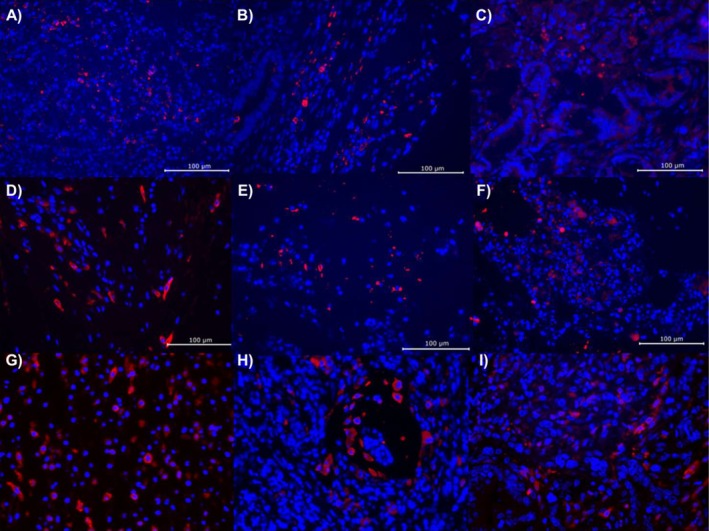
Immunofluorescent staining of CD206 (A–C), CD163 (D–F), and CD68 (G–I) found no significant differences between treatment groups in either males or females. Staining from control DW (A, D, G), GcL (B, E, H), and SsoPox (C, F, I) is shown.

To determine the physiological effects of QQ treatments that may be driving tumor growth and metastasis, endpoint fecal concentrations of IgA, calprotectin, and Lcn‐2 were tested but found to not be significantly different by treatment group in both male (ANOVA *F* = 0.126, 1.308, 0.771, *p* = 0.881, 0.291, 0.478) and female (ANOVA *F* = 2.078, 2.140, 0.711, *p* = 0.151, 0.144, 0.503) mice (Table 1). Similarly, none of the seven SCFAs measured had significantly different concentrations by treatment groups in either males or females (ANOVA *F* < 1.109, *p* ≥ 0.349, Table 2). Tumor tissue staining for CD206, CD163, and CD68, which are used to measure the abundance of immune infiltrates, including T cells and both M1 and M2 macrophages, also did not find significant differences by treatment group in either sex (Figure 4). While not conclusive, these findings suggest that the use of QQ enzyme treatments does not elicit a strong immune or inflammatory response. Similarly, few correlations were noted between the microbiome and ELISA or SCFA data. A higher relative abundance of *Duncaniella* was correlated with lower valeric acid levels in male mice (Spearman *ρ* = −0.597, *p* = 0.017). In female mice, relative abundances of *Muribaculaceae* were correlated, and *Alistipes* were inversely correlated with propionic acid concentrations (Spearman *ρ* = 0532, −0.555; *p* = 0.010, 0.007).

**TABLE 1:** Fecal concentrations (mean ± standard deviation, ng/mg) of IgA, calprotectin, and Lcn‐2 among male and female mice at student endpoint.

Analyte	DW	GcL	SsoPox	*F*	*p*
Female IgA	11.31 ± 1.26	14.97 ± 5.43	13.08 ± 1.29	2.078	0.151
Male IgA	3.53 ± 1.64	3.71 ± 2.80	3.81 ± 1.15	0.126	0.881
Female calprotectin	0.26 ± 0.69	0.96 ± 0.92	0.92 ± 0.42	2.140	0.144
Male calprotectin	0.17 ± 0.54	0.65 ± 0.71	0.36 ± 0.62	1.308	0.291
Female Lcn‐2	0.11 ± 0.06	0.38 ± 0.55	0.37 ± 0.63	0.711	0.503
Male Lcn‐2	0.08 ± 0.05	0.06 ± 0.02	0.07 ± 0.04	0.771	0.478

*Note:* Differences among treatment groups were evaluated by ANOVA.

**TABLE 2:** Fecal concentrations (mean ± standard deviation, μg/g) of short‐chain fatty acids among male and female mice at study endpoint.

Sex	Treatment group			ANOVA	
Male	DW	GcL	SsoPox	*F* (M)	*p* (M)
2‐Methylbutyric acid	8.21 ± 1.62	9.63 ± 5.01	10.03 ± 2.91	0.570	0.579
Acetic acid	875.80 ± 275.99	697.52 ± 150.21	874.98 ± 225.30	0.848	0.450
Butyric acid	177.90 ± 83.46	208.79 ± 45.79	194.40 ± 56.15	0.270	0.767
Isobutyric acid	15.67 ± 5.27	16.18 ± 10.67	18.33 ± 5.85	0.219	0.806
Isovaleric acid	10.88 ± 1.75	10.28 ± 5.97	13.87 ± 4.71	1.097	0.363
Propionic acid	234.16 ± 90.17	227.24 ± 102.60	264.28 ± 73.11	0.242	0.789
Valeric acid	20.00 ± 6.00	20.75 ± 5.42	24.15 ± 12.73	0.362	0.703

Female	DW	GcL	SsoPox	*F* (*F*)	*p* (*F*)
2‐Methylbutyric acid	11.70 ± 5.98	12.05 ± 7.95	10.40 ± 3.57	0.143	0.867
Acetic acid	815.87 ± 162.22	698.75 ± 176.93	700.85 ± 201.49	1.009	0.382
Butyric acid	380.19 ± 148.76	342.46 ± 138.66	321.87 ± 83.00	0.375	0.692
Isobutyric acid	20.16 ± 9.10	21.19 ± 12.22	18.13 ± 6.31	0.193	0.826
Isovaleric acid	15.55 ± 6.92	18.00 ± 12.88	14.74 ± 5.67	0.261	0.773
Propionic acid	155.50 ± 46.35	199.57 ± 86.00	197.67 ± 41.07	1.109	0.349
Valeric acid	24.92 ± 11.83	28.75 ± 12.37	25.25 ± 10.03	0.276	0.761

## 4 Discussion

Our results demonstrate that QQ treatment can have a significant effect on CRC metastatic rate, though we have yet to fully elucidate a likely mechanism of action. The overall gut microbiome compositional diversity was not affected by QQ treatment, as we hypothesized, but a lack of widespread categorization of bacterial species and the QS/QQ molecules they produce limited our ability to examine specific bacterial species that may have been directly impacted by GcL or SsoPox treatment. We hypothesized that QQ might impact immune responses and inflammation, which are known to be linked to the microbiota [39], as well as SCFA concentrations [53], as a measure of microbial metabolic function; however, our data suggest a limited impact of QQ on these processes.

Despite a lack of differences in immunity or metabolite production, QQ had a profound role in reducing metastasis among males while exacerbating it in females, suggesting a potential mechanism of action mediated by the host endocrine system. Although a consensus in the literature is lacking, higher levels of testosterone tended to be inversely correlated with CRC development in men but may be positively correlated in women [54, 55]. Interaction between the endocrine system and QS systems has been previously, but ambiguously, reported, suggesting a complex feedback loop potentially mediated by AHLs [56, 57]. Furthermore, previous studies found that women with polycystic ovary syndrome had decreased lactonase activity in their paraoxonase 1 enzyme, which is known to degrade QS signals [58] as well as raise testosterone levels and CRC risk [58, 59], further suggesting a potential connection between endocrine signaling, QS, and CRC. This may explain why female mice exhibited a severe adverse response to QQ treatment while male mice, who lacked sufficient estradiol to promote biofilm growth, did not.

Results of our study should be interpreted cautiously, as our experimental design uses a model of established, aggressive CRC and does not seek to study the role of QS/QQ in early CRC development. Future studies should explore the role of QS/QQ in tumor onset and progression through the use of sporadic and genetic models of CRC. Moreover, our study has several limitations, including a modest sample size, limited duration, and testing in only one genotype. Repetition with a higher sample size with sequential necropsies will be needed to determine the exact effects of QQ treatment on the rate of CRC metastasis. However, our current results highlight novel sex‐specific differences in response to distinct QQ lactonase treatments and suggest a potential interaction between host endocrine signaling, bacterial QS, and CRC metastasis. Unraveling the interplay between these complex systems will take decades of research but may unlock both novel therapies and substantial improvements to existing interventions. Our finding that these lactonase enzymes greatly impede metastatic progression from established and aggressive CRC suggests that QQ therapies may be a promising avenue for development, especially given the increasing frequency of earlier and more advanced disease in CRC.

## Author Contributions

**Matthew Dietz:** conceptualization; methodology; investigation; writing – original draft; data curation; formal analysis; validation; visualization; funding acquisition. **Travis J. Gates:** methodology; writing – review and editing; investigation; resources. **Rakesh Sikdar:** resources; writing – review and editing. **Subbaya Subramanian:** conceptualization; writing – review and editing. **Mikael H. Elias:** conceptualization; writing – review and editing; resources; project administration; funding acquisition. **Christopher Staley:** conceptualization; writing – review and editing; project administration; supervision; investigation; funding acquisition.

## Ethics Statement

The experiment was approved by the University of Minnesota Institutional Animal Care and Use Committee (IACUC), protocol 2212‐40606A.

## Conflicts of Interest

M.H.E. is a co‐founder, a former CEO, and an equity holder of Gene&GreenTK, a company that holds the license to WO2014167140 A1, FR 3068989 A1, FR 19/02834. M.H.E. has filed the patents EP3941206 and WO2020185861A1. M.H.E.'s interests with Gene&GreenTK have been reviewed and managed by the University of Minnesota in accordance with its conflict‐of‐interest policies. The remaining authors declare that the research was conducted in the absence of any commercial or financial relationships that could be construed as a potential conflicts of interest.

## Data Availability

Raw sequence data are deposited in the Sequence Read Archive of the National Center for Biotechnology Information under BioProject accession number SRP477359.
